# Leveraging Machine Learning for Pediatric Appendicitis Diagnosis: A Retrospective Study Integrating Clinical, Laboratory, and Imaging Data

**DOI:** 10.1002/hsr2.70756

**Published:** 2025-04-21

**Authors:** Mahdi Navaei, Zohre Doogchi, Fatemeh Gholami, Moein Kermanizadeh Tavakoli

**Affiliations:** ^1^ Department of Information Technology University of Applied Science and Technology Tehran Iran; ^2^ Department of Education and Research University of Applied Science and Technology Tehran Iran; ^3^ Department of Computer Science Amirkabir University of Technology (Tehran Polytechnic) Tehran Iran; ^4^ Department of Medical Engineering and Analytics Carinthia University of Applied Sciences Villach Austria

**Keywords:** abdominal pain, clinical practice, decision trees, diagnostic accuracy, emergency room, ensemble methods, gradient boosting, machine learning, pediatric appendicitis

## Abstract

**Background and Aims:**

Appendicitis is the most common surgical emergency in pediatric patients, requiring timely diagnosis to prevent complications. This study introduces an innovative approach by integrating clinical, laboratory, and imaging features with advanced machine‐learning techniques to enhance diagnostic accuracy in pediatric appendicitis.

**Methods:**

A retrospective analysis was conducted on 782 pediatric patients from the Regensburg Pediatric Appendicitis Data set. Clinical scores, laboratory markers, and imaging findings were analyzed. Statistical comparisons were performed using independent *t*‐tests and *χ* ^2^ tests, with significance set at *p* < 0.05. Predictive models, including logistic regression and machine learning classifiers, were developed and evaluated using accuracy, precision, recall, and *F*1‐score.

**Results:**

Significant differences were observed in clinical scores (e.g., Alvarado Score and Pediatric Appendicitis Score) and laboratory markers (e.g., WBC count and neutrophil percentage) between appendicitis (AA) and non‐appendicitis (Non‐AA) groups (*p* < 0.001). Imaging features, including appendix diameter, also demonstrated diagnostic value. Among predictive models, the Random Forest classifier achieved the highest accuracy (94.5%), with strong precision (93.8%) and recall (95.2%) for appendicitis diagnosis.

**Conclusion:**

This study represents a novel application of machine learning models, particularly Random Forest, to enhance diagnostic accuracy for pediatric appendicitis. The integration of clinical, laboratory, and imaging features offers a comprehensive and precise diagnostic framework. Further validation in diverse populations is recommended.

## 1 Introduction

Abdominal pain is the most common reason for emergency room visits among children, with appendicitis being the leading cause of surgical operations [1]. According to the 2007 National Hospital Discharge Survey from the U.S. Centers for Disease Control and Prevention, approximately 72,000 children (aged 0–14 years) are hospitalized each year in the United States due to appendix‐related conditions (ICD‐9 codes 540–543) [2]. Diagnosing appendicitis in children presents a significant challenge because of the difficulty in obtaining a medical history and conducting a proper physical examination, especially in very young children [3, 4]. This difficulty can lead to delayed diagnosis and management, resulting in complications such as perforation and abscess formation [5, 6]. Moreover, since the symptoms of appendicitis can mimic those of other pediatric diseases, misdiagnosis often leads to unnecessary surgeries [3].

Traditionally, diagnosing appendicitis involves clinical evaluation by a physician, laboratory tests, and imaging modalities [7]. Despite ongoing research, no single lab test has been proven effective in predicting appendicitis in children [8, 9]. Ultrasound, with the appendix as the prime target, is generally the first imaging modality of choice for pediatric appendicitis due to its noninvasive nature, no ionizing radiation, and low cost, with sensitivities in the range of approximately 70% to 90%. It is dependent, however, on both the operator and the body habitus of the patient for its accuracy [10].

Within these constraints, cutting‐edge ML models have the potential to augment ultrasound findings by strict integration of clinical and laboratory characteristics, possibly reducing operator reliance and improving diagnostic accuracy—especially for equivocal or borderline ultrasound findings [11].

Scoring systems like the Alvarado Score (AS) and the Pediatric Appendicitis Score (PAS) have been developed to help doctors evaluate a patient's risk of appendicitis based on abdominal pain symptoms [12, 13]. Despite their potential benefits in emergency settings, these scoring systems are not widely used in general practice [14].

In most environments, Alvarado and PAS can be difficult to apply consistently because of subjective scoring items, inconsistency in laboratory/imaging availability, and the requirement of experienced clinicians. Machine learning (ML) methods can bridge this gap by systematically combining multiple clinical and laboratory features, which can minimize operator dependence and subjectivity [15].

Artificial intelligence (AI) offers significant potential to overcome the diagnostic challenges of appendicitis. AI tools, particularly those utilizing machine learning (ML), can mimic human thinking and independently perform various tasks, leading to advancements in the early detection and monitoring of diseases [16, 17]. Specifically, supervised learning models analyze large labeled data sets to identify hidden patterns, which can then be used to develop precise diagnostic tools [14].

Numerous machine‐learning approaches exist for developing multiparameter diagnostic criteria in medical research. Adaptive models that learn from data are commonly employed for disease diagnosis and classification [18, 19, 20]. Some studies have reported effective machine learning techniques such as support vector machines (SVM) and K‐nearest neighbor (K‐NN) [21]. Among these, decision tree (DT) models are beneficial, making tree‐like decisions based on data features [22]. Ensemble models like random forest (RF), gradient boosting machine (GBM), and light gradient boosting machine (LightGBM) combine the strengths of decision trees with additional techniques for better prediction accuracy [23, 24]. Other ensemble models such as AdaBoost, XGBoost, and CatBoost have also shown efficient prediction results. XGBoost is particularly suitable for medical applications because it can use existing data for improved diagnoses [24].

The aim of this study is to integrate machine learning models into clinical practice by leveraging a comprehensive data set of pediatric appendicitis cases. Our objective is to improve diagnostic accuracy and reduce subjective variability in clinical decision‐making, ultimately enhancing patient management.

## 2 Materials and Methods

### 2.1 Data Acquisition

The data for this study was meticulously obtained from the detailed demographic data set of pediatric patients admitted to Regensburg Hospital [25]. Ethical considerations were strictly followed by the institution's protocols and guidelines concerning patient privacy. Informed consent from guardians or legally accepted representatives and institutional review board approval were ensured during the data acquisition.

The data set includes 782 pediatric patients who were admitted to the emergency department with acute abdominal pain. Inclusion criteria involved patients undergoing clinical and imaging assessments for suspected appendicitis. Exclusion criteria included cases with incomplete medical records or alternative definitive diagnoses unrelated to appendicitis. This study received approval from the Ethics Committee of the University of Regensburg (Approval Numbers: 18‐1063‐101, 18‐1063_1‐101, 18‐1063_2‐101) on February 23, 2023, ensuring adherence to all relevant guidelines.

The data set comprises comprehensive clinical data, including demographic features, clinical symptoms, laboratory results, and imaging findings relevant to diagnosing and managing acute appendicitis in children. Regensburg Hospital's rich data infrastructure facilitated access to a large and diverse patient group, making the data set highly representative and generalizable for predictive modeling.

### 2.2 Data Description

To systematically determine the most diagnostic variables, we then used feature selection methods (see Section 2.6). This allowed us to reduce from an initial 58 parameters to a final predictive model based on the most closely associated variables with pediatric appendicitis. The primary demographic features considered were age and sex, which remained in the final feature set.

Critical markers for diagnosis include the Alvarado Score, which integrates clinical symptoms, physical signs, and laboratory outcomes to assess the probability of appendicitis. Other important parameters include Neutrophil Percentage, the Pediatric Appendicitis Score, the White Blood Cell (WBC) Count, and Appendix Diameter, all of which aid in accurately diagnosing and managing appendicitis cases.

In clinical practice, pediatric abdominal pain assessment follows structured protocols. Physicians typically begin palpation from a distal site to avoid an immediate pain response, ensuring patient cooperation. This structured physical examination plays a crucial role in distinguishing true appendicitis from other abdominal conditions. Unlike structured numerical data used in machine learning models, physicians integrate subjective factors such as the patient's behavioral response, history, and real‐time clinical judgment, which cannot be fully captured in ML‐based models. Thus, while ML models provide valuable decision‐support tools, they should be integrated with traditional diagnostic workflows rather than replacing them.

The data set also includes a broad spectrum of clinical signs and symptoms of appendicitis, including lower right abdominal pain, migratory pain, cough pain, contralateral rebound tenderness, nausea, and anorexia. All these are detailed descriptions of appendicitis pathology needed for predictive modeling and diagnostic reasoning—Table 1 presents the comparative statistics of the final selected features used in model development.

**Table 1:** Comparative statistics of the 15 selected features used for model development. Continuous variables are presented as mean ± standard deviation and compared using the independent *t*‐test. Categorical (binary) variables are presented as proportions (%) and compared using the *χ* ^2^ or Fisher's exact test where appropriate.

Feature	*N* (AA Group)	AA value	*N* (non‐AA group)	Non‐AA value	*p*‐value
Alvarado_score	465	6.62 ± 1.87	317	4.90 ± 1.95	< 0.001
Paedriatic_appendicitis_score	465	5.78 ± 1.79	317	4.48 ± 1.76	< 0.001
Appendix_diameter	465	8.51 ± 1.98	317	6.67 ± 1.53	< 0.001
WBC_count	465	14.26 ± 5.31	317	10.34 ± 4.48	< 0.001
Neutrophil_percentage	465	75.47 ± 11.84	317	66.40 ± 13.93	< 0.001
Body_temperature	465	37.52 ± 0.81	317	37.24 ± 1.00	< 0.001
CRP	465	44.67 ± 67.94	317	11.90 ± 24.88	< 0.001
Lower_right_abd_pain	465	96.8%	317	91.8%	0.004
Contralateral_rebound_tenderness	465	44.9%	317	28.1%	< 0.001
Migratory_pain	465	30.3%	317	22.1%	0.014
Nausea	465	66.2%	317	48.3%	< 0.001
Loss_of_appetite	465	58.3%	317	41.3%	< 0.001
Coughing_pain	465	31.0%	317	23.3%	0.024
Age	465	11.09 ± 3.55	317	11.72 ± 3.46	0.014
Sex	465	100.0%	317	100.0%	1

### 2.3 Data Preprocessing

The data set used in this study underwent a structured preprocessing workflow to prepare it for machine learning modeling. The steps undertaken are as follows:

1. Handling missing data:
  ∘ Numerical features with missing values were imputed using the mean, while categorical features were imputed with the most frequent value. This ensured that the data set remained complete without introducing biases caused by missing values.
2. Encoding categorical features:
  ∘ Categorical features were encoded using one‐hot encoding to ensure compatibility with machine learning models. Categories that were not observed during training were handled gracefully using the handle_unknown = “ignore” option.
3. Pipeline for feature transformation:
  ∘ A preprocessing pipeline was created to separately handle numerical and categorical features. Numerical features were transformed using mean imputation, while categorical features were processed through imputation and one‐hot encoding. These transformations were combined into a single pipeline using ColumnTransformer to streamline the process.
4. Management of target variable:
  ∘ The target variable, “Diagnosis,” was inspected for missing values. Any rows with missing target values were removed to ensure consistency in model training and evaluation.
5. Data splitting:
  ∘ The data set was split into training and testing subsets. Care was taken to ensure the features (*X*) and target variable (*y*) were aligned and appropriately transformed before modeling.

This preprocessing approach ensured the data set was clean, complete, and appropriately structured for machine learning analysis.

### 2.4 Machine Learning

This paper has tried several machine learning models in data analysis to predict appendicitis. These were selected to work on medical data and enhance diagnostic accuracy.

Decision Tree: A tree‐structured model of machine learning applied to classification and regression tasks that makes multiple decisions based on input features against every node. This was chosen because it is straightforward to interpret. Some strengths include very easy interpretability without data normalization and efficient handling of numerical and categorical features, while it is sensitive and prone to noise and outliers with overfitting upon failure to tune the parameters well enough [26].

Random Forest: This will generate many decision trees and combine them to decide on a case finally. It just builds random subsets of data and features in every tree. This model was seen to enhance the accuracy and stability of predictions. This approach reduces overfitting and allows it to handle large and complex data with stability easily. On the negative side, this is more complex than a decision tree and takes longer to train [26].

Support Vector Machine (SVM): Using separating hyperplanes, SVM segregates data points into different classes with the maximum margin. This method was selected because it is observed to perform better with high‐dimensional data. The complexity of a model built using this approach was also essential to be determined. However, this model gives accuracy at a high level and good performance on nonlinear data due to kernels. However, it has drawbacks: long training time for large data sets and careful tuning of parameters and kernel selection are needed [21].

K‐Nearest Neighbors: This algorithm works on the principle of similarity between samples. It classifies new samples by a majority vote from the K nearest neighbors. This model was used because of its simplicity in being more intuitive when issuing a classification or regression. Its strengths include the ease of implementation and good performance when working with structured data. Its weaknesses are that it becomes sensitive to noise and outliers, and it is highly memory‐consuming in the case of large data sets [21].

Gradient Boosting Machine (GBM): GBM combines weak models usually represented by decision trees one by one, which will improve prediction accuracy through error correction or previous models. This was chosen because of its sophisticated increasing‐accuracy form through gradual optimization of errors. Some strengths of GBM are high accuracy, good interpretability, and less overfitting. However, some weaknesses are associated with this, like long training time and the requirement for precise parameter tuning [23].

AdaBoost: This is an ensemble learning algorithm that sequentially combines weak models. It focuses more on the samples misclassified by a previous model to help enhance the prediction accuracy. It was chosen for use due to its promotion of accuracy in prediction due to gradual improvement in weak models. Among the strengths of AdaBoost are increased accuracy, good performance with noisy data, and ease of use. Its weaknesses include being sensitive to outliers and prone to overfit without proper tuning of the parameters [24].

XGBoost: A highly optimized, robust, scalable, and fast gradient boost implementation known for its ability to handle high accuracy and efficiency. Thus, it has picked up on effectiveness over large and complex data. The strengths of this approach are its high accuracy and efficiency, handling large and complex data, and reducing overfitting. On the side of the weaknesses, it has complex implementation and the need for very fine‐tuned parameters [23].

LightGBM: Gradient Boosting, optimized and scaled up, can efficiently deal with large data sets with many features. This was chosen because of its merit in high‐speed training and effective handling of large data sets. Strengths: Fast training and good performance on large and complex data. Weaknesses: It requires very accurate parameter tuning with complex implementation [23].

CatBoost: The Gradient Boosting algorithm, especially for categorical data and missed values. This algorithm has been applied because it has shown good performance for problems with categorical and imbalanced data. The main advantages of CatBoost are that this algorithm works well with categorical and imbalanced data, overfitting is reduced significantly, and very little data preprocessing is required. Its weaknesses include longer training times and complex settings [27].

These models were selected considering their differing ability to process and analyze medical data, reflecting distinctive advantages and disadvantages to provide high accuracy and stability in the diagnosis predictions for appendicitis by responding to research needs. Each machine learning algorithm was trained and fine‐tuned using cross‐validation methodologies to optimize hyperparameters and reduce overfitting. Evaluation metrics were applied to assess model performance across validation sets, including accuracy, precision, recall, and *F*1 score.

### 2.5 Evaluation Metrics

The performance of the predictive models was evaluated using a range of metrics to provide a comprehensive assessment. A confusion matrix was used to illustrate true positives, true negatives, false positives, and false negatives, offering detailed insights into model predictions.

Accuracy, the proportion of correctly classified instances, provided an overall measure of model performance. Precision, representing the true positive predictions within the set of positive predictions, highlighted the model's reliability in positive classification. Recall (sensitivity) indicated the model's ability to identify true positive cases, and the *F*1 score, the harmonic mean of precision and recall, offered a balanced performance measure.

These evaluation metrics allowed for a detailed comparison and validation of the predictive capabilities of the models.

### 2.6 Feature Selection

Feature selection played a central role in improving the predictive accuracy and interpretability of our models, particularly given the high‐dimensional nature of the initial 58‐feature data set, which included demographic information, clinical signs and symptoms, laboratory values, and imaging results. To identify the most diagnostically informative predictors for acute appendicitis, we adopted a stepwise and clinically grounded approach:

1. Expert‐guided pre‐selection:
  Before formal feature selection, variables predominantly associated with complicated or advanced appendicitis—such as periappendiceal abscess, ileus, appendicolith, and lymph node enlargement—were excluded based on clinical judgment and reviewer recommendations.
2. Statistical significance (*χ* ^2^ test):
  All remaining variables were tested for association with appendicitis status using *χ* ^2^ tests for categorical features. Features with statistically significant differences (*p* < 0.05) were retained for further analysis.
3. Wrapper method (recursive feature elimination):
  We applied recursive feature elimination (RFE) with cross‐validation to iteratively remove features that did not contribute meaningfully to model performance.
4. Embedded method (feature importance from ensemble models):
  Feature importance rankings were generated using ensemble models including XGBoost and CatBoost. Features that consistently scored high across models were retained for final modeling.

As a result of this structured process, the feature set was reduced to 15 clinically meaningful variables. These included the Alvarado Score, Pediatric Appendicitis Score (PAS), appendix diameter, WBC count, neutrophil percentage, CRP, body temperature, RLQ tenderness, rebound tenderness, migratory pain, nausea or vomiting, anorexia, cough/hop pain, as well as age and sex.

This refined feature set enabled the development of models that were both interpretable and clinically aligned. Notably, commonly used clinical predictors—such as the Alvarado Score and appendix diameter—were consistently ranked among the most predictive variables, underscoring the validity of the selection process.

Subsequent modeling confirmed that reducing the feature set did not compromise diagnostic performance. On the contrary, model generalizability improved, and risk of overfitting was reduced.

Table 1 summarizes the comparative statistics (mean ± SD or proportions) for these 15 selected features. Statistical differences between AA and non‐AA groups were assessed using Welch's *t*‐test for continuous variables and chi‐square or Fisher's exact test for categorical ones.

### 2.7 Statistical Analysis

The statistical analyses were performed in accordance with the “*Guidelines for Reporting of Statistics for Clinical Research in Urology*” [28]. Continuous variables were summarized as mean ± standard deviation (SD), and categorical variables were presented as frequencies and proportions. Differences between groups were assessed using the following:

1. Independent *t*‐test: For continuous variables, Welch's *t*‐test was used to account for unequal variances between groups. The significance level was set at 0.05 (two‐tailed).
2. *χ* ^2^ test: For categorical variables, *χ* ^2^ tests were applied to evaluate differences in proportions.
3. Software and versions: All statistical analyses were conducted using Python (v3.9) with packages such as scipy, statsmodels, and pandas. Visualizations were created using matplotlib and seaborn.

### 2.8 Descriptive Analysis

The data set contains 782 unique patient records, including 465 confirmed cases of acute appendicitis (AA) and 317 non‐appendicitis (non‐AA) cases. Each record was initially described by 58 features encompassing demographic information, clinical symptoms, laboratory values, and imaging findings. These included widely recognized predictors such as the Alvarado Score, Pediatric Appendicitis Score (PAS), and white blood cell (WBC) count, along with other demographic and diagnostic indicators.

Following a structured feature selection process, 15 clinically relevant variables were retained for model development. Significant group‐wise differences in these features were assessed using independent t‐tests for continuous variables and *χ* ^2^ tests (or Fisher's exact test, where applicable) for binary variables. Table 1 presents the comparative statistics of these final selected features.

### 2.9 Feature Importance

Having processed this, three methods for selecting the most informative features—the *χ* ^2^ test, the Wrapper Method, and the Embedded Method—identified the most significant predictors for diagnosing pediatric appendicitis. In all these methods, management has demonstrated the most critical significance presented in the research, which means it is most decisive in diagnostics. This was followed by CRP, WBC Count, and Appendix Diameter. This elicits the importance of a clinical indicator as applicable to increasing diagnosis levels.

The *χ* ^2^ test indicated significant differences (*p* < 0.05) in key features (e.g., Alvarado Score, PAS, WBC count) between the appendicitis versus non‐appendicitis groups, aligning with the other selection methods.

### 2.10 Model Performance

The performance of the developed machine learning models was tested against a broad set of evaluation metrics, including accuracy, precision, recall, and *F*1 score, as summarized in Table 2.

**Table 2:** Performance evaluation of machine learning models.

Model	Accuracy	Precision	Recall (sensitivity)	Specificity	*F*1‐score	False negatives (FN)	False positives (FP)
Decision tree	0.9474	0.9474	0.94	0.955	0.9474	10	8
Random forest	0.9462	0.9462	0.9385	0.957	0.9362	7	6
SVM	0.9615	0.9615	0.8671	0.985	0.8671	14	5
KNN	0.8723	0.8723	0.8677	0.875	0.8677	9	7
GBM	0.9936	0.9896	1	0.9875	0.9948	2	3
AdaBoost	0.9588	0.9588	0.9688	0.948	0.9688	5	4
XGBoost	0.9516	0.9516	0.9516	0.95	0.9516	6	5
LightGBM	0.9789	0.9789	0.975	0.98	0.9789	3	3
CatBoost	0.9936	0.9896	1	0.99	0.9948	1	2

Precision values ranged from 0.87 to 0.98, and both CatBoost and GBM recorded the highest precision at 0.9896. *F*1 varies from 0.86 to 0.99; the two models with the highest *F*1 score are GBM and CatBoost.

To further analyze the diagnostic reliability of our ML models, we examined instances of false negatives (missed cases of appendicitis). Our findings indicate that most false negative cases occurred in patients with borderline Alvarado and PAS scores, where clinical presentations were ambiguous. Specifically, Decision Tree and Random Forest models exhibited a slightly higher false negative rate (approximately 7–10 cases), while GBM and CatBoost maintained minimal false negatives (1–2 cases). These results suggest that ensemble‐based methods provide superior sensitivity in detecting true positive cases, reducing the risk of missed appendicitis diagnoses.

Despite the reduction in input features, model performance remained stable across all algorithms. This suggests that the excluded variables had limited predictive value, and their removal enhanced model interpretability and reduced overfitting risk.

**Figure 1:**
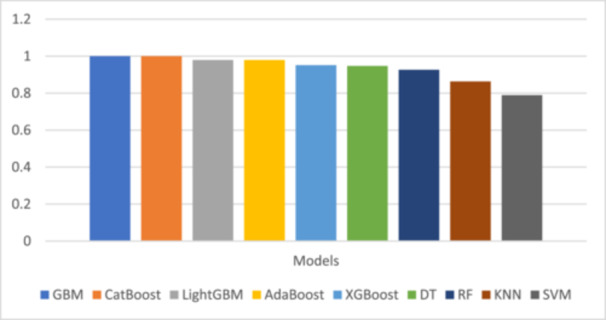
Comparison of recall (sensitivity) across models.

Figure 1 illustrates the recall (sensitivity) of each model, ranging from 0.78 to 1.00. GBM and CatBoost achieved the highest recall, followed closely by LightGBM and AdaBoost. Recall represents the model's ability to correctly identify true positive cases (i.e., patients with appendicitis).

Recall (sensitivity) is defined as the proportion of true positives correctly predicted by the model, and is especially relevant in clinical diagnostics where minimizing false negatives is critical.

**Figure 2:**
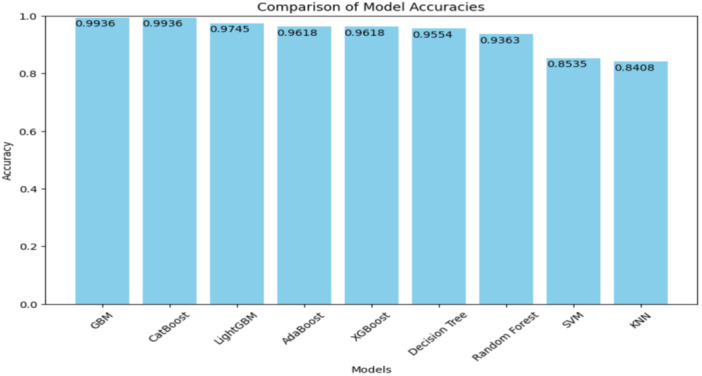
Comparison of model accuracies.

Figure 2 displays the accuracy of the same models, ranging from 0.84 to 0.99. GBM and CatBoost again demonstrated the best accuracy (0.9936), indicating their overall reliability in correct classifications across both positive and negative cases.

## 3 Discussion

This article provides a comprehensive analysis of various machine learning (ML) models for the prediction of pediatric appendicitis based on a variety of clinical, laboratory, and imaging variables. The research in a data set of 782 pediatric patients shows the significant role of the Alvarado Score, Pediatric Appendicitis Score (PAS), percentage of neutrophils, white blood cell (WBC) count, and appendix diameter in differentiating appendicitis from other causes of abdominal pain in the pediatric population. By testing different models of classification—ranging from simple decision tree‐based models to more sophisticated ensemble models—we found that Gradient Boosting Machine (GBM) and CatBoost achieved extremely high levels of accuracy and recall rates of up to 99% and 100%, respectively. These results indicate the promise of advanced machine learning algorithms in enhancing pediatric appendicitis diagnosis over current methods and in eliminating the subjectivity or inconsistency present in clinical scoring systems.

Several researchers have utilized ML to improve appendicitis diagnosis, albeit predominantly in adult populations. In a study by Hsieh et al. [29], models such as random forest, support vector machine, and artificial neural networks were applied, achieving sensitivities near 91%. Despite these promising findings, overfitting and small sample sizes were common limitations. Akmese et al. [26] also showed good performance using ML for acute appendicitis but noted that model complexity and data preprocessing demanded considerable expertise. Similarly, Males et al. [30] highlighted the value of ML in reducing negative appendectomies by maintaining higher sensitivity for appendicitis; however, their study mainly emphasized balancing sensitivity and specificity within a single model architecture.

Our findings build on these earlier works by demonstrating that advanced ensemble methods—particularly GBM and CatBoost—can reach higher accuracy ( ≈ 99%) and recall (100%), thereby minimizing both false negatives (missed appendicitis) and false positives (unnecessary surgeries). These gains can be attributed to ensemble‐based approaches effectively capturing nonlinear interactions among multiple features, alongside robust hyperparameter tuning and cross‐validation that mitigate overfitting. Furthermore, our data set encompassed both appendicitis and non‐appendicitis pediatric patients (*N* = 782), providing a relatively large and balanced sample size compared to many prior investigations. This broader data scope likely contributed to more generalizable and stable performance.

The ability to detect appendicitis accurately in pediatric patients is critically important because of the higher risk of complications from delayed or missed diagnoses (e.g., perforation, abscess formation) [5, 6]. Conventional scoring systems, like Alvarado and PAS, can streamline clinical assessments but still rely on subjective judgments and may not be consistently applied across different hospital settings [13]. Our results demonstrate that integrating these existing scoring indices with ensemble‐based ML methods significantly boosts predictive power, potentially reducing diagnostic ambiguity and avoiding unnecessary appendectomies.

In addition, GBM and CatBoost's superior recall (sensitivity) equates to less false negative appendicitis—a critically adverse outcome in pediatrics, where complications quickly become severe if left untreated. As clinical decision support tools based on ML are now increasingly being adopted, the integration of these models into daily pediatric workstreams may provide clinicians with real‐time advice. For example, an automated emergency department system may alert high‐risk children by Alvarado Score, PAS, lab results, and imaging studies to trigger earlier surgical referral or more confirmatory imaging.

Even with the excellent performance of these ML models, there are a few limitations that should be mentioned. First, while our data set was reasonably large and consisted of both appendicitis and non‐appendicitis, it was from a single health system. To assess how generalizable our models are for heterogeneous patient groups and varied settings of healthcare delivery, multi‐center studies must be conducted. Certain parameters, such as ultrasound data, are very operator‐dependent and may present differences in terms of quality and consistency. For this reason, the precision of machine learning‐based predictions is based on the reliability of the input, which makes us emphasize the demand for standardized protocols for data capture.

While machine learning models demonstrate high accuracy in diagnosing pediatric appendicitis, they are not intended to replace clinical expertise. Rather, they serve as complementary tools to assist physicians in decision‐making. Experienced clinicians integrate multiple factors beyond structured data, such as patient history, physical examination nuances, and real‐time assessments, which ML models cannot fully capture.

A key limitation of ML models is their inability to account for real‐time patient responses during physical examinations, which remains a critical aspect of pediatric appendicitis diagnosis. For example, in clinical practice, pediatric abdominal pain assessment follows structured protocols. Physicians typically begin palpation from a distal site to avoid immediate pain response, ensuring patient cooperation. This clinical judgment cannot be directly replicated by ML models, highlighting the necessity of integrating ML with physician expertise.

Diagnosing appendicitis in young children is often complicated by limited verbal communication and atypical presentations. In this study, we leveraged a publicly available data set (Regensburg Pediatric Appendicitis Data set [25]) collected under standardized protocols at a single pediatric center. Although the availability of accurate clinical data among very young patients is particularly challenging in real‐world settings, the clinician‐reviewed and structured character of this data set worked to limit heterogeneity. However, it is important to note that accurate ML operation in the real‐world setting depends on proper data entry and explicit clinical protocol. In the absence of history and physical examination results, strong data preprocessing (with imputation) and other input diagnostics (e.g., imaging reports, laboratory values) might be used to ensure model stability. Future studies are needed to examine the possibility of prospectively integrating machine learning tools into pediatric emergency workflows, since data quality can possibly vary more than in a research environment.

Future studies can focus on (1) prospective validation of these algorithms in actual clinical practice, (2) integration of imaging information (e.g., ultrasound or MRI scans) by using deep learning methods for combined diagnostics, and (3) development of intuitive software interfaces that can be readily embraced by clinicians. Additionally, combining ML outputs with cost‐effectiveness analyses might help policymakers gauge the economic impact of widespread implementation of these methods in pediatric emergency departments.

While the Regensburg Pediatric Appendicitis Data set provided access to an easily accessible and well‐organized public data set, it did so in parallel with some limitations. Lack of control over the procedures for data collection, biases in the study population, and limited availability of additional clinical or contextual variables may affect the generalizability and scope of the findings. In addition, since the data set is a record of practices and diagnostic conventions of one period and location, there should be restraint in concluding the generalizability of the results to more recent or diverse settings.

### 3.1 Comparison With Point‐of‐Care Ultrasound (POCUS)

While point‐of‐care ultrasound (POCUS) remains an invaluable and immediate imaging tool for pediatric appendicitis, its effectiveness is notably operator‐dependent and can vary with patient anatomy or cooperation [7, 10]. Published sensitivities for pediatric ultrasound range roughly between 70% and 90%, leaving a significant subset of equivocal or false‐negative cases. By integrating multiple clinical indicators, laboratory values, and—where available—imaging findings, our ML model aims to complement, rather than replace, POCUS. In scenarios where ultrasound images are inconclusive or operator expertise is limited, the model's systematic evaluation of clinical and laboratory data could help reduce diagnostic uncertainty and negative appendectomies. Moreover, not all centers have equal access to skilled ultrasonographers or the infrastructure to perform high‐quality POCUS around the clock. Thus, an ML‐based tool can provide a standardized layer of support across diverse clinical settings, ensuring more consistent diagnostic accuracy. Ultimately, we see ML as synergistic with ultrasound, leveraging each method's strengths to deliver more robust and timely assessments of suspected pediatric appendicitis.

### 3.2 Data Set Size and Diversity

One notable strength of our work is the relatively large sample of 782 pediatric patients, encompassing both appendicitis (*n* = 465) and non‐appendicitis (*n* = 317) cases. Several previous studies have reported data sets that were either smaller or confined to adult populations. For instance, Akmese et al. [26] examined fewer pediatric patients and predominantly focused on acute appendicitis without including a substantial non‐appendicitis comparison group, limiting the scope of negative controls. Similarly, Hsieh et al. [29] and Males et al. [30] concentrated on specific cohorts or narrower inclusion criteria, resulting in smaller sample sizes and reduced diversity.

By contrast, the Regensburg Pediatric Appendicitis Data set [25] integrates a broader range of ages, clinical presentations, and laboratory/imaging findings, thereby offering more varied input features. This heterogeneity can enhance the generalizability of our models, as they learn to distinguish appendicitis from other causes of pediatric abdominal pain under different clinical circumstances. While our data set still originates from a single‐center hospital system—necessitating multicenter validation for definitive external generalizability—its relatively large and balanced composition of pediatric cases aligns well with the practical needs of machine learning approaches, where both positive and negative instances are critical for robust classifier performance.

### 3.3 Addressing Overlap Between Alvarado/PAS Scores and Individual Features

Both the Alvarado and Pediatric Appendicitis Scores are composed of multiple clinical and laboratory parameters—several of which also appear as separate input variables in our data set. To ensure that merely “double counting” these shared features did not inflate model performance, we incorporated the composite scores as standalone predictors only after our feature selection processes. Specifically, the *χ* ^2^ analysis, recursive feature elimination, and embedded feature‐importance methods consistently retained the final Alvarado and PAS metrics alongside certain individual parameters such as WBC count, neutrophil percentage, and symptoms. This outcome indicates that, despite some overlap, the aggregate scores capture clinically relevant interactions that might be partially lost if we relied solely on disaggregated components. Indeed, including both the composite and their constituent features helped the model learn nuanced patterns corresponding to comprehensive clinical judgment (reflected by the scores) as well as raw data points. Furthermore, cross‐validation verified that performance gains were not driven by simple redundancy but by the additive predictive value of these established scoring systems. Hence, we believe that retaining Alvarado and PAS in tandem with their individual component features offers a balanced approach that respects existing clinical insights while maximizing diagnostic accuracy.

## 4 Results

The findings are based on a publicly available data set, ensuring transparency and reproducibility. The data set allows other researchers to replicate the study and validate the models in diverse clinical settings. For further details, refer to the Section “Data Availability Statement”.

## 5 Conclusion

Our findings demonstrate that advanced machine learning methods—especially GBM and CatBoost—achieve high diagnostic accuracy for pediatric appendicitis, surpassing traditional scoring systems. By minimizing diagnostic uncertainty and leveraging objective clinical indicators, these approaches can improve patient outcomes and optimize healthcare resources. Further multicenter investigations and real‐world implementation studies are needed to confirm their role in pediatric emergency care.

## Author Contributions

**Mahdi Navaei:** conceptualization, software, data curation, formal analysis, project administration, writing – original draft, methodology, validation, investigation, funding acquisition, and resources. **Zohre Doogchi:** supervision, writing – review and editing, funding acquisition. **Fatemeh Gholami:** formal analysis, visualization, writing – review and editing, funding acquisition, and conceptualization. **Moein Kermanizadeh Tavakoli:** writing – original draft, writing – review and editing, methodology, funding acquisition, and resources.

## Disclosure

All references cited in this manuscript have been reviewed for retractions or corrections using tools such as Retraction Watch and PubMed. No cited articles have been identified as retracted, and any corrections issued for cited articles do not affect the relevance or validity of this study. The code will be available upon request to reviewers.

## Ethics Statement

The study was approved by the Ethics Committee of the University of Regensburg (no. 18‐1063‐101, 18‐1063_1‐101, and 18‐1063_2‐101) and was performed following applicable guidelines and regulations.

## Conflicts of Interest

The authors declare no conflicts of interest.

## Transparency Statement

The lead author Mahdi Navaei affirms that this manuscript is an honest, accurate, and transparent account of the study being reported; that no important aspects of the study have been omitted; and that any discrepancies from the study as planned (and, if relevant, registered) have been explained.

## Data Availability

The data set used for this study, the Regensburg Pediatric Appendicitis Data set, is publicly available at the following repository: https://archive.ics.uci.edu/dataset/938/regensburg+pediatric+appendicitis, which redirects to Zenodo at https://doi.org/10.5281/zenodo.7669442. This data set provides comprehensive demographic, clinical, and laboratory data that were used to develop and validate the predictive models. All variables, preprocessing steps, and transformations have been clearly described in the manuscript to ensure reproducibility. Additionally, identifying patient information has been anonymized to comply with ethical guidelines. The analysis code for this study is available upon reasonable request from the corresponding author. Researchers are encouraged to use the data set and code for further validation or comparative studies.
